# A review of biomarker and imaging monitoring to predict heart failure recovery

**DOI:** 10.3389/fcvm.2023.1150336

**Published:** 2023-04-06

**Authors:** Crystal Lihong Yan, Luanda Grazette

**Affiliations:** ^1^ Department of Medicine, Jackson Memorial Hospital, University of Miami, Miami, FL, United States; ^2^ Division of Cardiovascular Medicine, University of Miami Health System, Miami, FL, United States

**Keywords:** heart failure recovery, reverse remodeling, predictors, biomarker monitoring, imaging monitoring, heart failure

## Abstract

Heart failure is a clinical syndrome caused by structural cardiac abnormalities that lead to increased intracardiac pressures and decreased cardiac output. Following cardiovascular insult or direct myocardial injury, neurohormonal activation triggers hemodynamic changes and cardiac remodeling to preserve cardiac output. While initially adaptive, cardiac remodeling eventually causes pathologic changes in cardiac structure that often compromise cardiac function. Reverse remodeling is the regression of abnormal cardiac chamber geometry and function after myocardial injury. In recent years, several classes of therapeutics have been associated with greater likelihood of reverse remodeling. Heart failure recovery and heart failure remission, terms encompassing the clinical correlates of reverse remodeling, have been associated with improved survival in patients with heart failure with reduced ejection. As such, identifying predictors of heart failure recovery can have important implications for guiding clinical practice and therapeutic innovation. This review addresses the role of biomarkers and imaging monitoring in predicting structural, functional, and clinical recovery in patients with acute and chronic heart failure.

## Introduction

Heart failure (HF) is a dynamic condition with varied clinical presentations and sequelae depending on factors such as etiology, concomitant conduction disease, pharmacologic management, and associated comorbidities (1, 2). For example, patients with heart failure with reduced ejection fraction (HFrEF) may progress to end-stage HF requiring advanced HF therapies or they may recover and be reclassified as patients with heart failure with recovered ejection fraction (HfrecEF) (3). Individuals with HF due to ischemic cardiomyopathy tend to have a worse prognosis due to fundamental differences in functional myocardial muscle mass from prior infarction compared with those with non-ischemic cardiomyopathy (4). In patients with HF and concomitant conduction disease, cardiac resynchronization therapy (CRT) significantly increases survival (5). Similarly, individual drugs of certain classes improve morbidity and mortality in HF patients, while others within the class do not (6). Given the heterogeneity in disease presentation and progression, identifying predictors of HF recovery has important implications for guiding clinical practice.

HF recovery can be defined in many ways. An essential process in countering HF progression is reverse remodeling or regression of the molecular, cellular, and tissue adaptions underlying cardiac remodeling. Cardiac remodeling describes the synchronized genomic, molecular, cellular, and interstitial changes that manifest as changes in size, geometry, and function of the heart following insult or injury. Reverse remodeling describes normative changes in size, geometry, and function of a previously “failing” heart. It is understood to occur following the removal of the triggering injury and the institution of therapeutic interventions which are thought to promote salutary molecular, cellular and interstitial changes. On the macroscopic level, reverse remodeling can restore normal cardiac chamber geometry and function after myocardial injury, a process often described as recovery or remission (4). However, precise criteria for reverse remodeling, recovery, and remission are yet to be completely defined or standardized by either parameters or thresholds. Some studies define reverse remodeling as a reduction in left ventricular (LV) volume or diameter, also described as structural recovery. Other studies use an LV ejection fraction (LVEF) increase by a certain percentage or above a certain threshold, which is described as functional recovery (7). Mortality reduction and improved function are well-recognized as critical clinical outcomes in HF. Reverse remodeling/structural recovery is associated not only with improved myocardial functionality, but it is also associated with improved survival (8, 9). As such, either structural, functional, or clinical recovery will be considered HF recovery in this review.

Many studies have sought to determine prognostic indicators of reverse remodeling and clinical outcomes. Baseline clinical parameters, biomarkers, and imaging findings, such as non-ischemic cause, female sex, and baseline LVEF, show strong predictive value (10–16). While helpful, baseline considerations reflect a single timepoint and, therefore, cannot be used in real-time to track or predict disease progression to modify or personalize treatment plans. This review focuses on the role of serial biomarker measurements (Table 1) and imaging as predictors of structural, functional, and clinical HF recovery over time.

**Table 1:** Studies evaluating biomarker monitoring to predict structural, functional, and/or clinical recovery in HF patients.

Author, year	Study population, *n*	Study design	Biomarker	Recovery
Cho, 2018 (17)	Acute HF, 175	Prospective cohort	NT-proBNP	Functional
Daubert, 2019 (18)	Chronic HFrEF, 116	Retrospective data from a RCT (GUIDE-IT)	NT-proBNP	Clinical Functional Structural
Januzzi, 2019 (19)	Chronic HFrEF, 654	Prospective cohort	NT-proBNP	Functional Structural
Weiner, 2013 (20)	Chronic HFrEF, 116	Retrospective data from a RCT (PROTECT)	NT-proBNP	Functional Structural
Zile, 2016 (21)	Chronic HFrEF, 1942	Retrospective data from a RCT (PARADIGM-HF)	NT-proBNP	Clinical
Felker, 2012 (22)	Acute HF, 685	Retrospective data from a RCT (ASCEND-HF)	TnI	Clinical
Felker, 2015 (23)	Acute HF, 1074	Retrospective data from a RCT (RELAX-AHF)	hsTnT	Clinical
Gaggin, 2014 (24)	Chronic HF, 150, 151	Retrospective data from a RCT (PROTECT)	hsTnT, sST2	Clinical Functional Structural
Masson, 2012 (25)	Chronic HF, 5284	Retrospective data from RCTs (Val-HeFT and GISSI-HF)	hsTnT	Clinical
Miller, 2009 (26)	Chronic HF, 150	Prospective cohort	TnT	Clinical
Motiwala, 2015 (27)	Chronic HFrEF, 99	Retrospective data from a RCT (PROTECT)	hsTnI	Clinical Functional Structural
Sato, 2001 (28)	Chronic HF, 60	Prospective cohort	TnT	Clinical Functional Structural
Wallenborn, 2017 (29)	Acute HFrEF, 456	Retrospective data from a RCT (INH)	hsTnI	Clinical Functional Structural
Anand, 2014 (30)	Chronic HFrEF, 1094	Retrospective data from a RCT (Val-HeFT)	sST2	Clinical
Boisot, 2008 (31)	Acute HF, 150	Prospective cohort	ST2	Clinical
Broch, 2012 (32)	Chronic HFrEF due to ICM, 1449	Retrospective data from a RCT (CORONA)	sST2	Clinical
Miller, 2016 (33)	Chronic HFrEF, 180	Prospective cohort	sST2, galectin-3	Clinical
O’Meara, 2018 (34)	Chronic HFrEF, 1758	Retrospective data from a RCT (PARADIGM-HF)	sST2	Clinical
Anand, 2013 (35)	Chronic HFrEF, 1097	Retrospective data from a RCT (Val-HeFT)	Galectin-3	Clinical
Lok, 2012 (36)	Chronic HF, 182	Prospective cohort	Galectin-3	Clinical Structural
Motiwala, 2013 (37)	Chronic HFrEF, 145	Retrospective data from a RCT (PROTECT)	Galectin-3	Clinical Functional Structural
Van der Velde, 2013 (38)	Acute and chronic HF, 1653	Retrospective data from RCTs (CORONA and COACH)	Galectin-3	Clinical
Weir, 2013 (39)	Acute myocardial infarction and HFrEF, 100	Retrospective data from a RCT	Galectin-3	Structural

HF, heart failure; HFrEF, heart failure with reduced ejection fraction; hsTnI, high sensitivity troponin I; hsTnT, high sensitivity troponin T; n, number of participants included in the relevant primary analysis; RCT, randomized controlled trial; sST2, soluble suppression of tumorigenesis-2; TnI, troponin I; TnT, troponin T.

## Biomarker predictors

### NT-proBNP

Natriuretic peptides, namely B-type natriuretic peptide (BNP) and N-terminal pro-B-type natriuretic peptide (NT-proBNP), are well-established HF biomarkers and indicate myocyte stretch and wall stress. Pro B-type natriuretic peptide is the precursor protein that is cleaved into equimolar concentrations of biologically active BNP and inert NT-proBNP. NT-proBNP circulates in higher plasma concentrations compared to BNP since the former primarily relies on passive renal excretion, whereas the latter is additionally cleared by peripheral receptors and enzymatic breakdown (18, 20). The conversion ratio of NT-proBNP to BNP depends on factors such as atrial fibrillation, age, and renal function but on average is around 6.25:1 (19). Studies evaluating the use of natriuretic peptides for serial monitoring of HF recovery have primarily utilized NT-proBNP and it is unknown whether the results can be extrapolated to BNP. Reduction in NT-proBNP has been associated with greater reverse remodeling and improved clinical outcomes in patients with HFrEF.

Reduction in NT-proBNP over time is a predictor of improved LV volumes and function. The PROTECT study aimed to determine whether NT-proBNP guided HF management to a NT-proBNP goal of <1,000 pg/ml was associated with reverse remodeling as evaluated by echocardiography. 151 participants were randomized to NT-proBNP guided vs. standard of care (SOC) HF management. Both groups saw improved echocardiographic parameters of cardiac structure and function with pharmacologic treatment. However, the effect sizes on all measures of LV volumes and function, some measures of diastolic function and estimated filling pressures, and all measures of right ventricular function were larger in the NT-proBNP guided therapy group compared to the SOC group (17). By the same token, the GUIDE-IT Echo substudy evaluated the effect of achievement of an NT-proBNP goal of <1,000 pg/ml on cardiac structure and function in 116 patients. Patients who achieved the NT-proBNP goal had significantly reduced LV volumes, increased left ventricular ejection fraction (LVEF), and improved global longitudinal strain (GLS) compared with patients who did not reach the NT-proBNP goal. The greater the absolute decrease in NT-proBNP level, the greater the impact on left ventricular volumes (21). Published in the same year as the GUIDE-IT Echo substudy, the PROVE-HF study showed that reduction in NT-proBNP after sacubitril-valsartan use was weakly correlated with improved cardiac structure and function at 6- and 12-months follow-up. There were 654 study participants who completed the study. Interestingly, most of the NT-proBNP reduction was observed 2 weeks after medication initiation when most patients received the lowest dose. Significant improvement in left-sided cardiac volumes and LVEF on echocardiography was observed as early as 6 months and the effect continued until 12 months (28). While the previously mentioned studies followed patients at clinic visits over a period of months, one study looked at NT-proBNP change during one hospitalization and found that NT-proBNP change >1633.5 pg/ml from admission to discharge was a predictor of LV functional recovery (27).

The structural changes associated with NT-proBNP reduction appear to translate into improved clinical outcomes. In the GUIDE-IT Echo cohort, patients who achieved the NT-proBNP goal also had a significant reduction in a composite endpoint of death and HF hospitalization after 12 months. However, it is important to note that the patients who failed to achieve the NT-proBNP goal were significantly more likely to have ischemic heart disease and other comorbidities that may have confounded the results (21). Patients from the PARADIGM-HF trial who had a reduction in NT-proBNP also had lower rates of cardiovascular mortality and HF hospitalization and this relationship was independent of the treatment group (29). These studies, taken together, demonstrate that serial NT-proBNP measurements can help predict structural, functional, and clinical recovery in HF patients.

### Troponin

Cardiac troponins (cTn) are well-established markers of myocyte injury and are commonly used to diagnose ischemic cardiovascular events. In patients with chronic HF, persistently elevated troponin concentrations are associated with adverse remodeling and increased mortality (22, 24). While the increase in cTn over time is consistently associated with worse clinical outcomes in HF patients, cTn change appears to be an inconsistent predictor of reverse remodeling.

Studies evaluating cTn change based on the categorization of serial measurements show that HF patients with decreased or persistently low cTn levels at follow-up have significantly improved structural and functional recovery compared to those with increased or persistently high cTn levels. An early study performed in 60 patients with idiopathic dilated cardiomyopathy categorized patients based on their troponin T (TnT) pattern (TnT <0.02 ng/ml throughout the study, TnT ≥0.02 ng/ml initially and then <0.02 ng/ml during follow-up, TnT ≥0.02 ng/ml initially and during follow-up). Patients with TnT <0.02 ng/ml during follow-up had significantly decreased left ventricular diastolic dimension (LVDd) and increased LVEF compared to those with TnT at or above the threshold. However, there was no significant correlation between changes in TnT and changes in LVDd or LVEF (24). Post hoc analysis from the Interdisciplinary Network Heart Failure (INH) study examined hsTnI change as a prognostic marker during the transition from hospitalization for acute decompensated heart failure (ADHF) to chronic heart failure. 456 of the original 875 patients were included in the study. Baseline hsTnI levels measured before discharge and follow-up hsTnI levels measured after 6 months were categorized into tertiles. Patients had hsTnI decrease by at least one tertile (37%), increase by at least one tertile (11%), or remain in the same tertile (52%). At the 6-month follow-up, patients in all tertiles had significant improvement in LVEF. However, LV end-diastolic diameter and systolic tricuspid valve gradient only improved in patients who had hsTnI decrease to or remain in the lowest tertile, suggesting a possible interplay between the degree of myocyte injury and the capacity for cardiac reverse remodeling (23).

Studies evaluating cTn change in HF patients based on time spent in response relative to thresholds show a weak or no effect on cardiac reverse remodeling. Post hoc analyses from the PROTECT trial have examined the predictive value of serially measured high-sensitivity troponins for LV reverse remodeling in patients with HFrEF (22, 26). Participants were scheduled for clinic visits every 3 months up to 12 months, with blood samples collected and frozen at each visit for future biomarker measurement. In one analysis using high-sensitivity troponin I (hsTnI) concentration, 99 of the original 151 patients were included. The median hsTnI concentration for all subjects at baseline (10.9 pg/ml) was used as a threshold to assess the relative percentage of time spent at or below the threshold. While there was no significant correlation with the absolute or relative change in LV volumes, time spent with hsTnI ≤10.9 pg/ml was weakly correlated with the absolute and relative change in LVEF (22). On the other hand, another analysis using a previously defined hsTnT threshold (14 pg/ml) in 150 of the original 151 patients found no significant relationship between time spent at or below threshold and LVEF, LV end-systolic volume index, or LV end-diastolic volume index (26). The difference in results between the two analyses may be explained by the different biomarker thresholds used or by the lack of adjustment for significant differences between groups in the former analysis, as patients with higher troponin levels were older and had worse HF symptoms, worse renal function, and higher baseline NT-proBNP concentrations (22, 26).

Change in cTn is a predictor of clinical outcomes in patients with acute and chronic HF. In three of the previously described studies, patients with persistently high TnT concentrations had increased mortality rates (24); patients with greater time in hsTnI response had decreased incidence of cardiovascular events (22); and patients with high follow-up hsTnI levels had increased cardiovascular re-hospitalization (23). Serial TnI levels were measured on admission and again 48–72 h after in 685 of the original 7,141 ADHF participants in the ASCEND-HF trial. Relative TnI increase ≥20% was a significant predictor of 30-day mortality. Additionally, 48–72 h TnI level was a better predictor of 30-day mortality than baseline TnI level (25). The RELAX-AHF trial was also conducted in patients hospitalized with ADHF. HsTnT levels were measured in the 1,074 participants on admission and days 2, 5, and 14. Higher peak hsTnT and greater peak change were significantly associated with increased 180-day cardiovascular mortality (31). In the INH cohort, patients in whom hsTnI increased or remained in the highest tertile (13%) at 6 months post-discharge had the highest one-year cardiovascular hospitalization rate. In contrast, patients in whom hsTnI decreased or remained in the lowest tertile (60%) had better clinical outcomes (23). A prospective cohort study including 150 HF patients routinely measured TnT levels every 3 months over 2 years. Ambulatory patients with more frequent or persistent troponin elevation had a higher risk of death and cardiac transplantation (32). Lastly, 5,284 chronic HF patients from the Val-HeHF and GISS-HF trials had hsTnT levels measured at randomization and after 4 or 3 months, respectively. Patients whose hsTnT increased above the upper limit of normal (13.5 ng/l) had an increased risk of mortality compared to those whose hsTnT remained below the cutoff. Furthermore, follow-up hsTnT was a slightly better predictor than baseline hsTnT (30).

### Soluble St2

Soluble suppression of tumorigenesis-2 (sST2) is a less broadly utilized HF biomarker that reflects myocyte fibrosis and inflammation. However, SST2 change appears to be a reliable predictor of clinical outcomes in HF patients.

Increase in sST2 is a predictor of increased morbidity and mortality. A prospective study of 150 patients hospitalized for ADHF had blood samples collected daily up to 6 times between admission and discharge. Increased ST2 during hospitalization was associated with increased 90-day mortality (33). In a pre-specified substudy of the CORONA trial, which investigated the effect of rosuvastatin on HFrEF patients with ischemic cardiomyopathy, 1,449 of the original 5,011 participants were included who had sST2 measured at baseline and after 3 months. SST2 increase ≥15.5% over 3 months was weakly but significantly associated with increased cardiovascular death, nonfatal myocardial infarction, or stroke (34). The prognostic value of sST2 change was also examined in the Val-HeFT trial, which evaluated the addition of valsartan to standard HF therapy. 1,094 of the original 5,010 HFrEF patients had sST2 measured at baseline, 4 months, and 12 months. An increase in sST2 at 12 months was significantly associated with worse clinical outcomes (40). In the PROTECT cohort, an increase from sST2 ≤ 35 ng/ml to >35 ng/ml was associated with a significantly shorter time to first cardiovascular event, such as worsening HF, HF hospitalization, and cardiac death (26). Lastly, a study of 180 ambulatory HFrEF patients found that while change in sST2 level over time was not predictive, persistently elevated sST2 levels over time were prognostic in identifying increased risk of death or cardiac transplantation (36).

Decrease in sST2 has been associated with both improved and no impact on survival. In the previously mentioned cohort study of 150 ADHF patients, decreased ST2 predicted survival. Patients with ST2 decrease ≥15.5% during hospitalization had a 7% mortality rate, whereas those who did not achieve this threshold had a 33% mortality rate (33). Another study from the PARADIGM-HF trial evaluated 1,758 of the original 8,399 HFrEF patients. Serial sST2 measurements were collected at baseline, 1 month, and 8 months. Reductions in sST2 concentrations at 1 month were associated with reductions in cardiovascular death and HF hospitalization. Furthermore, changes in sST2 were linearly correlated with clinical outcomes, suggesting against the use of specific thresholds to evaluate this relationship (39). On the other hand, sST2 decrease was not associated with improved risk from the Val-HeFT trial. The degree of sST2 change also did not add further prognostic value beyond that of the follow-up value (40). This may be due to the fact that while sacubitril/valsartan was associated with sST2 reduction and improved clinical outcomes in PARADIGM-HF, valsartan in Val-HeFT was associated with attenuation of the rate of rise in sST2 compared with the placebo group over 12 months. This difference may be another example of the potentiating effects of neprilysin inhibition with angiotensin receptor blockade that are still to be fully recognized.

Studies examining the impact of sST2 change on structural and functional HF recovery are sparse. A *post hoc* analysis of the PROTECT cohort showed that greater time with sST2 ≤ 35 ng/ml predicted a decrease in LV end-diastolic index (26).

### Galectin-3

Galectin-3 is another marker of myocyte fibrosis and inflammation. It is unique from the previously described biomarkers in that baseline values have exhibited greater predictive value in heart failure with preserved ejection fraction (HFpEF) as opposed to HFrEF (37). High dose spironolactone (50 mg daily over 25 mg daily) has been shown to reduce galectin-3 concentrations (38). In contrast, valsartan did not lower galectin-3 levels (35). From the PROTECT study, no medication classes (beta blockers, angiotensin converting enzyme inhibitors, angiotensin receptor blockers, mineralocorticoid receptor antagonists, loop diuretics, or thiazide diuretics) significantly affected galectin-3 levels over time (41). Change in galectin-3 appears to be a poor predictor of structural recovery but a good predictor of clinical outcomes.

Change in galectin-3 does not appear to reflect change in LV volumes but may be associated with change in LVEF. Analysis of data from the DEAL-HF trial including 182 patients found no correlation between change in galectin-3 and change in LV volume at 3 or 12 months (42). Data from a randomized controlled trial of 100 patients designed to investigate the effect of eplerenone on reverse remodeling in HFrEF patients after acute myocardial infarction was also used to examine the relationship between galectin-3 and reverse remodeling over 6 months. Structural and functional cardiac parameters were assessed using cardiac magnetic resonance imaging. In line with findings from the DEAL-HF trial, there was no correlation between change in galectin-3 and change in any LV volume measurement (43). Not only was data from the PROTECT trial used to evaluate NT-proBNP, cTn, and sST2 monitoring, but it was also used to evaluate the utility of galectin-3 monitoring. Outcomes were assessed using the median baseline galectin-3 value (20 ng/ml) as a threshold. Neither baseline nor subsequent galectin-3 was associated with LV volume changes. However, a higher percentage of time with low galectin-3 (≤20 ng/ml) was associated with LVEF increase over time (41).

Galectin-3 increase appears to be a predictor of increased morbidity and mortality, but the converse may not be true. From the PROTECT trial, galectin-3 increase ≥15% was significantly associated with increased risk of cardiovascular events at 3, 6, and 9 months. Patients with persistently high galectin-3 levels had the most cardiovascular events, followed by low-to-high, high-to-low, and persistently low galectin-3 levels (41). Another study using data from two large trials (CORONA and COACH) had similar findings. A threshold advised by the US Food and Drug Administration (17.8 ng/ml) was used to divide patients into a low (<17.8 ng/ml) or high (>17.8 ng/ml) galectin-3 category. Patients with low-to-high galectin-3 had significantly increased all-cause mortality and HF hospitalization rates compared to patients with persistently low galectin-3. Conversely, patients with high-to-low galectin-3 had considerably decreased all-cause mortality and HF hospitalization rates compared to patients with persistently high galectin-3. Data analysis was also performed to corroborate these findings using percent change in galectin-3 levels. Galectin-3 increase ≥15% adjusted for baseline galectin-3 among other covariates was significantly associated with increased mortality risk and HF hospitalization compared with patients who stayed within 15% of their baseline galectin-3 level. Although galectin-3 decrease ≥15% was not associated with significant reduction in clinical outcomes (44). In addition to sST2 analysis, data from the Val-HeFT trial was used to evaluate galectin-3 use. Each 1 ng/ml increase in galectin-3 was associated with a 2.9% mortality rate increase at 4 months and 5.2% mortality rate increase at 12 months (35). On the other hand, change in galectin-3 levels was not a predictor of clinical outcomes in two previously mentioned studies (36, 42).

## Imaging predictors

### Echocardiography

Echocardiography is the first-line imaging modality for assessing cardiac chamber geometry and function. Myocardial deformation imaging is a newer echocardiographic technique using tissue Doppler-based or 2-dimensional speckle tracking-based methods to assess myocardial contractile function (45). Studies evaluating the use of serial echocardiography measurements to predict HF recovery are limited. A substudy of the EchoCRT trial included 614 of the original 809 HFrEF patients with narrow QRS width and ventricular dyssynchrony. Dyssynchrony was determined by echocardiographic findings of tissue Doppler-based longitudinal velocity delay ≥80 ms or speckle tracking-based radial strain delay ≥130 ms. Persistent or worsening dyssynchrony at 6-month follow-up was significantly associated with increased death and HF hospitalization, irrespective of CRT (46). A case report described the temporal pattern of strain parameters and LVEF over 5 time points in patients with HFrEF due to stress-induced cardiomyopathy. At HF presentation, the patient had a global longitudinal strain (GLS) of −6.2% and LVEF of 39%. Two days later, GLS improved to −10.7% with a relatively unchanged LVEF of 40%. Both GLS and LVEF eventually normalized at 3-month and 18-month follow-up. Early improvement in GLS preceded LV functional recovery, suggesting a potential role for GLS monitoring in patients with stress-induced cardiomyopathy to predict LV functional recovery (47).

Although not the focus of this review, GLS monitoring can detect and guide the management of chemotherapy-induced cardiotoxicity. Systematic reviews of echocardiography performed before, during, and after chemotherapy treatment support early reduction in GLS as a predictor of cardiotoxicity, defined as a decrease in LVEF or the development of HF (48, 49). A cohort study of 116 females with HER-2 positive breast cancer had patients undergo an echocardiogram at baseline and every 3 months to determine GLS and LVEF during chemotherapy treatment. A relative decrease in GLS by >15% of baseline was considered subclinical cardiac dysfunction, whereas LVEF reduction to <50% was considered overt HF. The use of GLS identified 27 patients with subclinical cardiac dysfunction when LVEF was still normal. These patients were started on concomitant beta-blockers and ACE-inhibitors, which allowed for the completion of chemotherapy without progression toward overt HF in 23 patients (50). By the same token, a randomized controlled trial of 307 patients treated with anthracycline-based chemotherapy compared GLS-guided vs. LVEF-guided initiation of cardioprotective treatment on LVEF at 12-month follow-up. Although there was no difference in LVEF between groups, the GLS-guided treatment arm had a significantly lower reduction in LVEF when only patients who received cardioprotective therapy were compared (51). Taken together, these findings suggest that GLS monitoring can be used to guide initiation of cardioprotective treatment during chemotherapy to prevent reduction in LVEF or discontinuation of cancer treatment. Studies evaluating the use of GLS monitoring to predict HF recovery are lacking.

### Cardiac magnetic resonance imaging

Compared to echocardiography, cardiac magnetic resonance (CMR) imaging has less technician-dependent variation and is not limited by poor acoustic windows, making it a useful alternative or adjunct imaging modality. Specific sequences such as late gadolinium enhancement and T1-weighted are used to assess the presence and degree of myocardial fibrosis (52). Studies of serial CMR measurements in predicting HF recovery are also lacking. The absence of late gadolinium enhancement (LGE) at baseline is a strong independent predictor of reverse remodeling and improved clinical outcomes (53–57). All patients with LGE at baseline had LGE at follow-up and no patients without LGE developed LGE at follow-up, suggesting against a role for serial evaluation of LGE to predict recovery (54). However, more studies are needed to draw any conclusions regarding the role of CMR monitoring in HF recovery.

## Conclusions

Reverse remodeling, recovery and remission are overlapping terms, sometimes used synonymously, that describe the regression of maladaptive cardiac structure and function alterations that occur in the setting of heart failure. NT-proBNP, cTn, sST2, and galectin-3 are cardiac biomarkers that may be useful in real-time to help presage the structural and functional changes associated with clinical recovery in heart failure patients and thus may help guide clinical management (Table 2, Central Illustration). Serial monitoring of NT-proBNP, cTn, sST2, and galectin-3 levels can help predict clinical outcomes in HF patients. Over time decreases in NT-proBNP (18, 21) and cTn (23, 24) are predictors of improved clinical recovery, while increases in cTn (22, 24, 26–29), sST2 (25, 30–32), and galectin-3 (38–40) over time are predictors of worse clinical recovery. Moreover, reduction in NT-proBNP is a strong predictor of structural and functional recovery (17–20). Whereas change in cTn (22–25) and galectin-3 (36–38) appear to be unclear and poor predictors of reverse remodeling, respectively. The role of sST2 monitoring in predicting reverse remodeling is promising but yet to be determined (25). Most published studies in imaging such as echocardiography and CMR have focused on the value of baseline measurements as predictive factors (53–60). The potential of serial imaging in predicting HF recovery is a subject for further inquiry. Given the strong association between reverse remodeling and improved clinical outcomes and survival, validated predictors of recovery could have great utility as clinically relevant intermediate surrogates. Accurate predictors of recovery could be beneficial in estimating prognosis, evaluating therapeutic efficacy, and optimizing care for individual patients.

**Table 2:** Summary of the utility of biomarker monitoring to predict structural, functional, and clinical recovery in HF patients.

Biomarker	Structural	Functional	Clinical
NT-proBNP	Yes	Yes	Yes
Troponin	Unclear	Unclear	Yes
sST2	Unclear	Unclear	Yes
Galectin-3	No	Unclear	Yes

NT-proBNP monitoring can be used to predict structural, functional, and clinical HF recovery. Troponin and sST2 monitoring can be used to predict clinical HF recovery, but their use to predict structural or functional HF recovery are unclear. Galectin-3 monitoring can be used to predict clinical HF recovery, does not predict structural HF recovery, and is unclear to predict functional HF recovery. HF, heart failure; sST2, soluble suppression of tumorigenesis-2.
